# Positive Psychological Intervention Delivered Using Virtual Reality in Patients on Hemodialysis With Comorbid Depression: Protocol and Design for the Joviality Randomized Controlled Trial

**DOI:** 10.2196/45100

**Published:** 2023-06-16

**Authors:** Rosalba Hernandez, Ken Wilund, Killivalavan Solai, David Tamayo, Drew Fast, Prasakthi Venkatesan, James P Lash, Claudia M Lora, Lizet Martinez, Geovana Martin Alemañy, Angela Martinez, Soonhyung Kwon, Dana Romero, Matthew H E M Browning, Judith T Moskowitz

**Affiliations:** 1 Department of Population Health Nursing Science College of Nursing University of Illinois Chicago Chicago, IL United States; 2 Department of Kinesiology and Community Health College of Applied Health Sciences University of Illinois at Urbana-Champaign Urbana, IL United States; 3 Center for Innovation in Teaching and Learning University of Illinois at Urbana Champaign Urbana, IL United States; 4 Division of Nephrology College of Medicine University of Illinois Chicago Chicago, IL United States; 5 School of Social Work University of Illinois at Urbana-Champaign Urbana, IL United States; 6 Department of Parks, Recreation and Tourism Management College of Behavioral, Social and Health Sciences Clemson University Clemson, SC United States; 7 Department of Medical Social Sciences Feinberg School of Medicine Northwestern University Chicago, IL United States

**Keywords:** virtual reality, hemodialysis, positive psychology, emotional well-being, mindfulness, depression, comorbid depression

## Abstract

**Background:**

Depression is highly prevalent in individuals on hemodialysis, but it is infrequently identified and remains undertreated. In this paper, we present details of the methodology of a randomized controlled trial (RCT) aimed at testing the feasibility and preliminary efficacy of a 5-week positive psychological intervention in individuals on hemodialysis with comorbid depression delivered using immersive virtual reality (VR) technology.

**Objective:**

We aim to describe the protocol and design of the *Joviality* trial whose main objectives are 2-fold: determine the feasibility of the *Joviality* VR software through metrics capturing rates of recruitment, refusal, retention, noncompliance, and adherence, as well as end-user feedback; and assess preliminary efficacy for outcomes measures of depressive symptoms, psychological well-being and distress, quality of life, treatment adherence, clinical biomarkers, and all-cause hospitalizations.

**Methods:**

This 2-arm RCT is scheduled to enroll 84 individuals on hemodialysis with comorbid depression from multiple outpatient centers in Chicago, Illinois, United States. Enrollees will be randomized to the following groups: VR-based *Joviality* positive psychological intervention or sham VR (2D wildlife footage and nature-based settings with inert music presented using a head-mounted display). To be eligible, individuals must be on hemodialysis for at least 3 months, have Beck Depression Inventory-II scores of ≥11 (ie, indicative of mild-to-severe depressive symptoms), be aged ≥21 years, and be fluent in English or Spanish. The *Joviality* VR software was built using agile design principles and incorporates fully immersive content, digital avatars, and multiplex features of interactability. Targeted skills of the intervention include noticing positive events, positive reappraisal, gratitude, acts of kindness, and mindful or nonjudgmental awareness. The primary outcomes include metrics of feasibility and acceptability, along with preliminary efficacy focused on decreasing symptoms of depression. The secondary and tertiary outcomes include quality of life, treatment adherence, clinical biomarkers, and all-cause hospitalization rates. There are 4 assessment time points: baseline, immediately after the intervention, 3 months after the intervention, and 6 months after the intervention. We hypothesize that depressive symptoms and hemodialysis-related markers of disease will substantially improve in participants randomized to the VR-based *Joviality* positive psychology treatment arm compared with those in the attention control condition.

**Results:**

This RCT is funded by the National Institute of Diabetes and Digestive and Kidney Diseases and is scheduled to commence participant recruitment in June 2023.

**Conclusions:**

This trial will be the first to test custom-built VR software to deliver a positive psychological intervention, chairside, in individuals on hemodialysis to reduce symptoms of depression. Within the context of an RCT using an active control arm, if proven effective, VR technology may become a potent tool to deliver mental health programming in clinical populations during their outpatient treatment sessions.

**Trial Registration:**

ClinicalTrials.gov NCT05642364; https://clinicaltrials.gov/ct2/show/NCT05642364

**International Registered Report Identifier (IRRID):**

PRR1-10.2196/45100

## Introduction

### Background

End-stage kidney disease (ESKD) requiring hemodialysis is a taxing condition that requires difficult self-care practices (eg, multiple weekly treatment center visits) [1]. Depression is the most pervasive psychological issue in this patient population and presents a substantial added burden: one-third of individuals on hemodialysis in the United States report compromised emotional well-being [2]. Comorbid depression exacerbates disease progression and is associated with increased markers of systemic inflammation; compromised cellular immunity [3]; and adverse clinical outcomes, including lower rates of treatment adherence [4], greater hospitalization risk, and shorter survival [5]. Possible mechanisms by which depression heightens morbidity and mortality in individuals on hemodialysis include negative effects on treatment adherence, impairment of the immune system, and hampering of healthy dietary practices [6].

Few individuals on hemodialysis experiencing psychological distress are currently being diagnosed and treated [7]. Pharmacotherapy has seen limited application, given the substantial pill burden among individuals on hemodialysis and reluctance by physicians given the possibility for drug-drug interaction and low medication adherence [8]. Health care providers and patients are reluctant to add another drug to the existing regimen unless severe psychopathology (eg, suicide ideation) is evident [8]. Despite the limited uptake of antidepressant medication in people on hemodialysis, nonpharmacologic strategies to boost psychological well-being continue to be underused and understudied [9,10].

The few published trials on efficacy of psychosocial strategies to improve depressive symptoms in individuals on hemodialysis overwhelmingly focus on face-to-face delivery of cognitive behavioral therapy [10]. Although cognitive behavioral therapy shows promise in reducing symptoms of depression and improved compliance of fluid intake [11,12], this approach presents significant challenges given the complexity and intensiveness needed for deployment, which requires highly trained and skilled clinicians for appropriate delivery and multiple in-person communications.

### Rationale for the Study

There is a critical need to identify practical, well-accepted, sustainable, and cost-effective strategies to reduce depressive symptoms and increase levels of psychological well-being in individuals on hemodialysis considered at risk. Fittingly, there has been a proliferation of programs that promote psychological assets and resilience, with the recognition of the importance of focusing on individual-level strengths and human flourishing—instead of solely focusing on negative psychological indicators, deficits, and emotional pathology [13,14]. Evidence shows that positive psychological assets (eg, optimism, positive affect, life meaning, and purpose) are uniquely related to better emotional and physical well-being, independent of the effects of negative emotion [15-21]. A meta-analysis of 25 positive strengths-based interventions targeting constructs such as gratitude, happiness, and optimism found a medium effect size for relief of depressive symptoms (median *r*=0.26), with stronger effects seen for phenotypes of participants with current depressive symptoms [22,23]. Published studies comparing positive psychology interventions with treatment as usual show effectiveness and durability similar to those of traditional psychotherapy or pharmacotherapy [22].

Accompanying expanded focus on strengths-based approaches is an increase in the use of technology to deliver these interventions that explicitly target boosting of positive emotions and factors of resilience. Testing remote distribution options (eg, mobile phone apps) for strengths-based interventions provides evidence of their efficacy and cost-effectiveness. Advantages of web-based delivery options include generally lower cost, easier dissemination, more consistent fidelity to the intervention protocol through automation that counteracts going off script, and greater accessibility in settings with Wi-Fi availability [24,25]. Comparative effectiveness trials document similar effect sizes for internet-based psychotherapy compared with traditional face-to-face approaches, with high patient satisfaction when rating these high-technology platforms [26,27]. Barriers to entry associated with traditional in-person forms of therapy may be bridged using web-based platforms that offer low-threshold and convenient at-home alternatives.

A participative technology that has rarely been used in individuals on hemodialysis is fully immersive 3D virtual reality (VR), although recent evidence in other clinical populations hints at its promise as an effective therapeutic tool [28]. VR is an evolving technology that presents an immersive experience to transport users to fictitious yet realistic and lifelike environments. Users wear a head-mounted display (HMD) or headset that recognizes physical motion (eg, head movement and remote control) to navigate VR (3D) worlds, which can replicate realistic settings or complex science fiction environments [29]. A meta-analysis confirms the utility of VR for pain reduction when pooling 11 trials that included individuals experiencing varied pain-inducing therapies or complaints (ie, burn injuries and wound redressing, postsurgical pain, and complex dental procedures, among others) [30]. This is the question that remains: “How might VR technology be used in individuals on hemodialysis to decrease procedural pain and discomfort and simultaneously deliver strengths-based programming to address the pervasive psychological distress experienced in this patient population?”

### Hypothesis and Objectives

To answer the question of the utility of VR in individuals on hemodialysis, we describe the design for a randomized controlled trial (RCT) that tests whether VR is a viable interface to deliver an evidence-based positive psychological intervention as a means to boost emotional well-being and reduce symptoms of depression in individuals on hemodialysis. VR has the benefit of allowing individuals on hemodialysis to digitally travel to multiple settings across the globe and briefly leave the confines of the clinic, which may offer further salutary benefits.

Specifically, we describe the design and protocol for testing our VR-based *Joviality* software, intended to decrease depressive symptoms and boost psychological well-being in individuals on hemodialysis. This study details the methodology of a prospective, 2-arm, parallel RCT that is set to launch in the June of 2023 and that has the following aims: to (1) evaluate the acceptability and feasibility of a 5-week positive psychological intervention, delivered using a VR platform, through the consideration of rates of recruitment, refusal, retention, noncompliance, and adherence, as well as end-user feedback; and (2) test the preliminary efficacy of our VR-based positive psychological intervention in comparison with that of an active control arm on outcomes of depressive symptoms, psychological well-being and distress, quality of life, treatment adherence, clinical biomarkers, and all-cause hospitalizations in individuals on hemodialysis at 3 and 6 months after the intervention. At trial conclusion, we expect that our software will have high system usability with superior performance, offering an enjoyable and positive end-user experience, and that participants in the *Joviality* treatment arm will show significant improvements in psychological well-being, treatment adherence, and hospitalization rates.

## Methods

### Overview and Study Design

This study describes the design and protocol of a 2-arm RCT, inclusive of both groups requiring use of an HMD: (1) VR-based *Joviality* positive psychological intervention, an immersive program that teaches 8 different skills to boost positive emotion with focused lessons on noticing of positive events, positive reappraisal, gratitude, acts of kindness, and mindful or nonjudgmental awareness, among others; and (2) sham VR [31], which consists of 2D wildlife and nature-based videos with inert background music. This prospective trial plans to recruit and enroll study participants from the University of Illinois Hospital Outpatient Dialysis Center, DaVita Kidney Care, and Nephrology Associates of Northern Illinois and Indiana. Both arms will be exposed to the virtual environments intraprocedurally during regular hemodialysis treatment sessions (ie, 3 times per week for 25- to 30-minute immersive sessions on each occasion). After the 6-month assessment period, participants in the control arm will be given access to the *Joviality* software.

Randomization procedures will use the web-based REDCap (Research Electronic Data Capture; Vanderbilt University) system, with 1:1 allocation across trial arms. Block randomization will occur among varying block sizes of 4, 6, and 8, with stratification by sex, depressive symptom severity, and recruitment site. The randomization procedure will be generated by our lead statistician (RD) and implemented by the project director (GM), who will actively participate in intervention delivery. However, the project director will not be involved in survey assessments to ensure safeguards against the risk of bias. Research staff conducting survey assessments will be blinded on assignment across intervention arms. Enrolled participants will also be blinded to randomization assignment, with a generic description of the VR content as *VR travel* and identification as either *group* *A* or *group* *B*.

### Ethics Approval

This VR-based trial has been approved by the institutional review board of the University of Illinois Urbana-Champaign and the University of Illinois Chicago (19190). Following requirements for an ethics approval process, all study-related documents (eg, recruitment flyers and consent forms) conform to specific language approved by the lead institutional review board, inclusive of its content, elements, and wording.

### Trial Registration and Guidelines

The trial has been registered a priori at ClinicalTrials.gov (NCT05642364). The clinical trial will follow published CONSORT (Consolidated Standards of Reporting Trials) guidelines, along with auxiliary standards of the CONSORT 2010 statement for social and psychological interventions (CONSORT-SPI 2018) [32].

### Staff Training, Data Collection, and Participation

Research staff and coinvestigators will be required to undergo human participants research training by completing the National Institutes of Health Human Research Protection Training module or the Collaborative Institutional Training Initiative program. Survey data will be collected using Health Insurance Portability and Accountability Act (HIPAA)–compliant server software, with each enrollee assigned a unique identifier to ensure privacy and confidentiality. All enrollees will provide written informed consent. Enrollees of both trial arms will be compensated for their time and participation. They will receive US $120 for participation in trial activities, specifically, for the completion of all survey assessments (US $30 for baseline assessment and US $30 for each follow-up measure).

### Sample Size

We estimated sample size requirements of a 2-arm RCT to evaluate whether our VR-based positive psychological intervention will result in clinically meaningful improvements in depressive symptoms compared with an active control. The following operational characteristics were assumed: (1) medium Cohen effect size of *d*=0.35, (2) a within-participant correlation of depressive symptoms over time (*P*=.60), (3) a 1-sided type I error probability of 0.10 [33], and (4) a statistical power of 85% [34]. This approach yields an estimated sample size requirement of 34 participants per study arm under equal allocation. Assuming an attrition rate of 20%, an updated estimation produces a sample size of 34 / 0.8 = 42 participants per study arm.

### Study Setting, Participants, and Eligibility

Trial participants must meet the following inclusion criteria: (1) on hemodialysis for at least 3 months before trial enrollment, (2) a Beck Depression Inventory-II (BDI-II) score of ≥11 [35], (3) age ≥21 years, (4) sufficient visual and audio acuity to enable immersion in the VR world, and (5) fluent in English or Spanish. Participation is restricted to individuals with a BDI-II score of ≥11 because this indicates elevated depressive affect and represents a group that may procure most benefit from the intervention (ie, greatest possible effect size) through targeting of psychological well-being and resilience. Women are eligible independent of pregnancy status. The exclusion criteria include (1) unavailable for study period; (2) cognitive impairment denoting dementia [36]; (3) diagnosis severely limiting life expectancy (eg, metastatic cancer); (4) physical limitations restricting use of an HMD (eg, facial injury); and (5) a history of epilepsy, seizures, or vertigo.

### Recruitment and Enrollment

Potentially eligible patients will be recruited in person by approaching them in the waiting room of the clinic before or during regularly scheduled hemodialysis sessions and at other suitable times during the patient consult or treatment period. Patients will also be remotely recruited through telephone calls made by research staff through contact information extracted from electronic medical records (EMRs). Research staff will also distribute flyers and brochures (chairside) to individuals on hemodialysis during regular clinic visits. Once full eligibility is determined and the patient expresses willingness to enroll in the trial, they will be provided a copy of the informed consent form for their signature. After submitting the signed informed consent form and before randomization, enrollees will complete the baseline assessment consisting of REDCap interviewer-led surveys.

### Intervention Arm: VR-Based Joviality Positive Psychological Intervention

The VR *Joviality* software architecture and content were built by the University of Illinois Urbana-Champaign Center for Innovation in Teaching & Learning, as led by the digital art director (KS) and principal investigator (RH). Adapted from the web-based course developed by Cohn et al [25], that is, *Developing Affective Health to Improve Adherence*, the original text-based curricula were transposed into a VR software system (ie, Unity [Unity Technologies]) using agile design principles, including brainstorming, storyboarding, designing, testing, and refining, thus transforming textual content into an interactive 3D environment. Finally, software design features have integrated an interface to easily modify the language in which content is presented (ie, English vs Spanish).

The 3D VR software, called *Joviality*, is a strengths-based positive psychological intervention that consists of a skills-based curricula to boost positive emotion and foster individual-level characteristics of resilience and grit. Our multicomponent 5-session intervention teaches the following eight skills: (1) noticing positive events, (2) amplifying positive events, (3) gratitude, (4) behavioral activation, (5) mindfulness, (6) positive reappraisal, (7) personal strengths, and (8) acts of kindness (Table 1). The skills are presented sequentially, starting with noticing of positive events in daily life and ending with the benefits of volunteerism and carrying out of acts of kindness. Only 1 new lesson is made available each week. Successive curricular content is unlocked only after all assigned weekly activities have been completed. Once the lessons are unlocked, participants can revisit prior lessons as a refresher or if they enjoyed a particular previous module.

Each skill is taught in a distinctive virtual environment using the Meta Quest Pro (October 2022 release date). The delivery of intervention content will require VR use for no more than 30 minutes during each hemodialysis session (ie, 30-minute sessions thrice weekly)—this will deter social isolation because patients will have ≥3 hours remaining to interact with neighboring patients and staff. VR immersion will only occur chairside when patients are already sedentary and in a seated position during regularly scheduled hemodialysis treatment. Table 1 details the weekly curricular content and the VR setting in which each lesson is delivered. As noted previously, end users on hemodialysis will be able to select whether the curricular VR content is presented in English or Spanish based on their preference and fluency.

**Figure 1:**
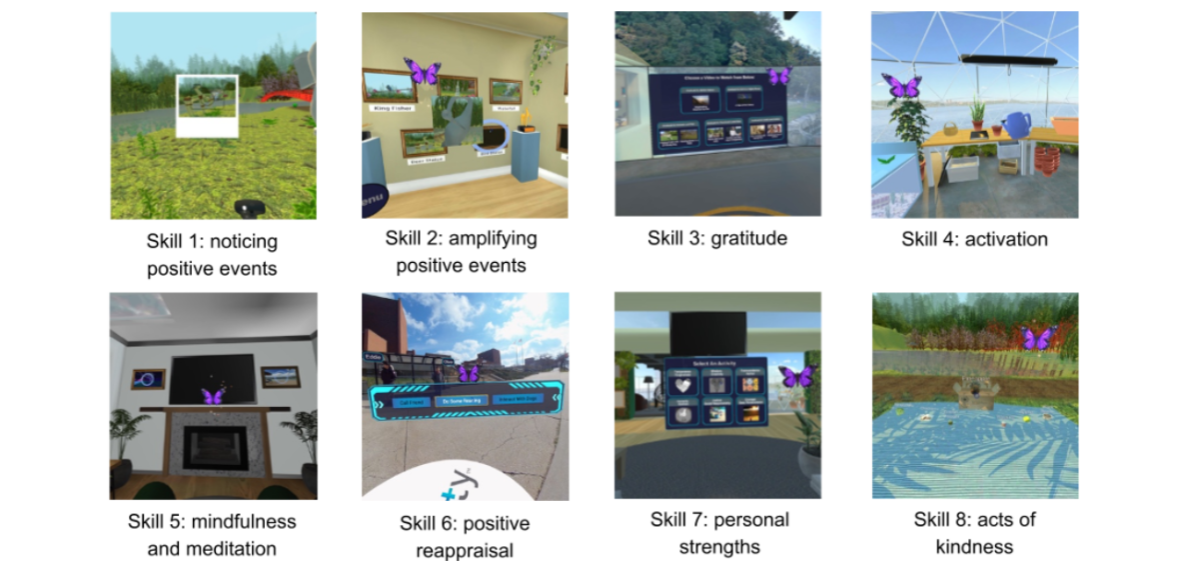
Scenes across Joviality modular lessons.

As part of *Joviality*, end users begin each lesson in a virtual lobby outfitted with lounge furniture, flatscreen television, and an instructional green wall where they sequentially select curricular lessons (Figure 1). Skill 1 takes place in a Japanese garden with lush greenery, wildlife, and a waterfall, where end users learn about positive events while snapping virtual photographs using head gaze movement. During skill 2, end users are transported to an art gallery where they participatively display the photographs they took while at the Japanese garden. The lesson on gratitude takes place in an outdoor patio where end users receive didactic instructions, view 2D videos augmenting curricular content, and visit 3D environments to appreciate nature and wildlife. Skills 4 and 5 are situated in a greenhouse, garden, or beach environment where end users play interactive games reinforcing lessons on behavioral activation, mindfulness, and meditation. The remaining lessons take place in the main lobby space. Across all lessons, VR environments present didactic instructions and interactive games and quizzes, along with 2D videos throughout.

**Table 1:** Joviality positive psychology curricular content and lessons.

Week and title			Description		Virtual reality environment
**Week 1**					
	Noticing positive events	Recognizing and focusing on positive events in life		Japanese garden	
	Amplifying positive events	Capitalizing on positive events and making the events more vivid and longer lasting		Museum gallery	
	Gratitude	Practicing thankfulness and appreciation of life, positive circumstances, and family or friends		Outdoor patio	
**Week** **2**					
	Activation	Breaking out of the harmful spirals that worsen emotions		Greenhouse	
**Week** **3**					
	Mindfulness and meditation	Paying attention and developing nonjudgmental awareness of one’s thoughts, feelings, and physical sensations in the present moment		Meditation garden and private beach	
**Week** **4**					
	Positive reappraisal	Understanding positive reappraisal and that reappraisal can substantially increase positive emotions in the face of stress, particularly when the silver lining is identified		Lobby and neighborhood bus stop	
	Personal strengths	Recognizing and enhancing a unique set of personal strengths, talents, skills, and positive qualities		Lobby	
**Week** **5**					
	Acts of kindness	Understanding the positive effects on mental and physical health when engaging in kind acts, volunteerism, and helping others		Lobby	

### Control Arm: Nonimmersive Sham VR

Participants randomized to the control arm will receive a rigorous placebo, based on the clinical trial guidelines of the VR Clinical Outcomes Research Experts panel [37], that consists of 2D nonimmersive visual content displayed on the HMD [28,38]. Footage of wildlife and nature-based settings are visually displayed as part of the sham VR with inert music that is unlikely to promote high levels of relaxation or distraction. The sham VR experience has very passive features such that it mimics viewing content on a large flatscreen television. Passive viewing during the sham VR experience rotates content using 20 different videos and has a duration time that is matched to that of *Joviality* over the 5-week intervention period. The delivery of the sham VR content will use the same VR hardware (ie, Meta Quest Pro) as that of the intervention arm to further promote the blinding of end users and research staff.

### Study Outcome Measures

**Figure 2:**
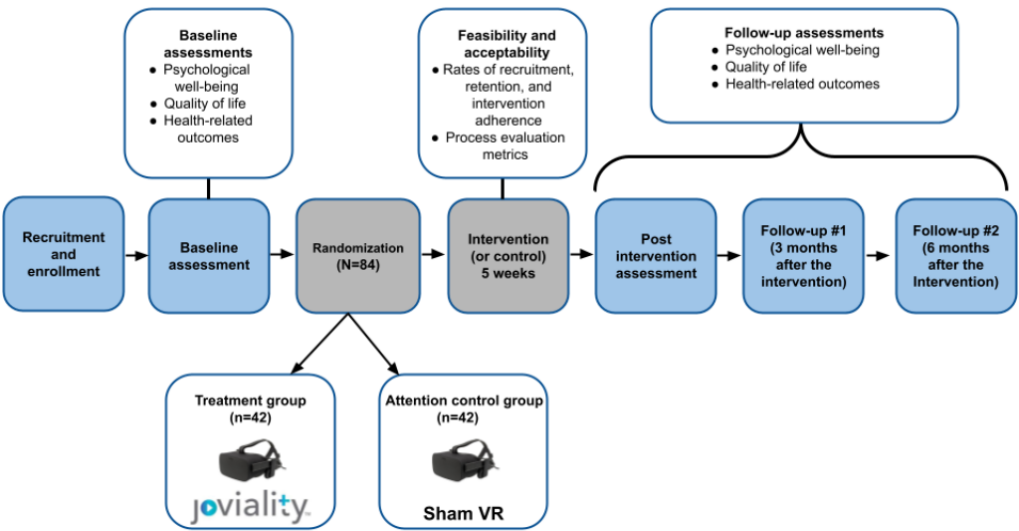
Joviality trial timeline and flowchart. VR: virtual reality.

The VR-based randomized trial has 4 assessment time points: baseline (T0), immediately after the intervention (T1; five weeks after baseline), follow-up 1 (T2; three months after the intervention), and follow-up 2 (T3; six months after the intervention). Figure 2 provides a visual of the design and timeline of survey measures. Staff collecting baseline and follow-up data (interview assessors), will be blinded to intervention assignment of enrollees and will not be involved in randomization procedures or any aspects of intervention delivery.

Table 2 details the primary, secondary, and tertiary outcomes of the RCT, along with process evaluation metrics. The primary outcome testing efficacy targets depressive symptoms, as measured by the BDI-II [35]. The PROMIS (Patient-Reported Outcomes Measurement Information System) [39,40] computer adaptive test for depressive symptoms will serve as an additional measure to triangulate the findings of the BDI-II. The central construct serving as our main secondary outcome is psychological well-being, inclusive of its hedonic, evaluative, and eudaimonic domains. Specific domains of psychological well-being will be assessed using PROMIS adaptive measures, including life satisfaction, positive affect, life meaning and purpose, and self-efficacy for managing emotions in the context of a chronic condition [39,40]. PROMIS measures have undergone psychometric testing and show high levels of reliability and validity [41]. Additional measures of psychological well-being include optimism using the 7-item State Optimism Measure [42]; emotional vitality, as measured using select items of the General Well-Being Schedule [43,44]; and the 10-item Flourishing Index, which broadly captures “a state in which all aspects [health and well-being] of a person’s life are good” [45]. PROMIS measures will also be used to assess anxiety levels [39,40], along with use of the Perceived Stress Scale [46].

The tertiary measures focus on quality of life, treatment adherence, and disease-related outcomes. First, we will assess self-reported quality of life using the 24-item Kidney Disease Quality of Life-Short Form [47]. Data extracted from the EMR, triangulated through interviews with enrollees or proxies, will be used to document the number of dialysis sessions missed and the total number of hospitalizations, with details on cause and length of stay. EMR data will also be used to capture the following biomarkers: urea reduction ratio, serum albumin concentration, blood urea nitrogen concentration, creatinine concentration, calcium phosphate product, and interdialytic weight gain (% Δ kg per day). We will also capture the etiology of chronic kidney disease via EMR data and self-reported information from enrollees.

Data on relevant covariates will be collected. These will include demographic factors, medical comorbidities [48], and medication use. The EMRs will be used to collect data on age, date of birth, sex, height, and weight. Surveys will be used to collect information on annual household income, educational attainment, marital status, health insurance status, employment, nativity status and number of years in the United States, and country of origin. The Charlson Comorbidity Index [48] will be used to extract codes of the International Classification of Diseases, Tenth Revision (ICD-10), from EMRs, inclusive of the following 18 conditions: myocardial infarction, congestive heart failure, peripheral vascular disease, cerebrovascular disease, dementia, chronic pulmonary disease, rheumatic disease, peptic ulcer, mild liver disease, diabetes without chronic complications, diabetes with chronic complications, hypertension, hypercholesterolemia, hemiplegia or paraplegia, any malignancy (including lymphoma and leukemia, except malignant neoplasm of the skin), moderate-to-severe liver disease, metastatic solid tumor, and AIDS or HIV. Medication use will be extracted by research staff through the review of medication lists using EMR data. Interview assessors will review the medication list with study participants to verify its accuracy and note any errors or inconsistencies.

We will also collect feasibility and process evaluation metrics (Table 3). Finally, we will extract information from the Meta Quest Pro across patient end users, inclusive of accelerometer data, eye tracking, and visual scanning of pupils, along with facial expressions.

**Table 2:** Joviality measures of feasibility and metrics testing efficacy.

Measure				Time frame		Type		Source
**Psychological well-being**								
	Beck Depression Inventory-II		In the past 2 weeks		Primary outcome		REDCap^a^	
	**PROMIS^b^ computer adaptive tests**							
		Depression	In the past 7 days		Secondary outcome		REDCap	
		Anxiety	In the past 7 days		Secondary outcome		REDCap	
		General life satisfaction	N/A^c^		Secondary outcome		REDCap	
		Positive affect	In the past 7 days		Secondary outcome		REDCap	
		Life meaning and purpose	N/A		Secondary outcome		REDCap	
		Self-efficacy for managing chronic conditions—managing emotions	Right now		Secondary outcome		REDCap	
	Perceived Stress Scale		In the last month		Secondary outcome		REDCap	
	State Optimism Measure		Right now or present moment		Secondary outcome		REDCap	
	Emotional vitality (via General Well-Being Schedule)		In the last month		Secondary outcome		REDCap	
	Flourishing Index		In the past days		Secondary outcome		REDCap	
Quality of life: 24-item Kidney Disease Quality of Life-Short Form				N/A		Tertiary outcome		REDCap
**Health-related outcomes**								
	Interdialytic weight gain (% Δ kg per day)		N/A		Tertiary outcome		EMR^d^ or MyChart (EPIC Systems Corporation)^e^	
	Dialysis sessions missed		N/A		Tertiary outcome		EMR or MyChart (EPIC Systems Corporation)	
	All-cause hospitalizations		N/A		Tertiary outcome		EMR or MyChart (EPIC Systems Corporation)	
	Kidney-related biomarkers: urea reduction ratio, serum albumin concentration, blood urea nitrogen concentration, creatinine concentration, calcium phosphate product, and hemodialysis etiology		N/A		Tertiary outcome		EMR or MyChart (EPIC Systems Corporation)	
**Feasibility and process evaluation: VR^f^ utility and usability**								
	Simulator Sickness Questionnaire		N/A		Feasibility		REDCap	
	Igroup Presence Questionnaire		N/A		Feasibility		REDCap	
	System Usability Scale		N/A		Utility		REDCap	

^a^REDCap: Research Electronic Data Capture (Vanderbilt University).

^b^PROMIS: Patient-Reported Outcomes Measurement Information System.

^c^N/A: not applicable.

^d^EMR: electronic medical record.

^e^EPIC: Electronic Health Record for the Integrated Care Team.

^f^VR: virtual reality.

**Table 3:** Joviality trial metrics of feasibility and acceptability.

Measure	Description	Target
Recruitment and refusal	Measured by the proportion of potential enrollees who were approached for recruitment but decided not to enroll	N/A^a^
Retention	Defined as completing all postintervention assessments, categorized as a binary outcome (yes or no), %	≥75
Adherence	Measured by the proportion of the intervention completed (eg, number of virtual reality environments viewed out of the total possible available across skill lessons in the intervention), %	≥75
Acceptability	Measured by whether a participant would recommend Joviality to other individuals on hemodialysis (ranging from 0=definitely not to 10=definitely yes)	≥8.0

^a^N/A: not applicable.

### Planned Analyses

#### Aim 1: Software Testing and Quality Assurance

Before deploying the *Joviality* VR software among patient end users, the software will be tested by the digital art director (KS) and visual media designer (DF) to ensure that the experience aligns with the original intentions of the research team and to ferret out accessibility or usability concerns. The software will also be tested for performance to ensure that the equipment can run the software without dropping frames or lagging, which can lead to motion or simulation sickness in participants on hemodialysis. User testing will also inform software editing. End users will have the option to experience the final content and audio of the VR environment in English or Spanish.

#### Aim 2: Acceptability and Feasibility

Key metrics to assess acceptability and feasibility are as follows: (1) recruitment rates, (2) refusal rates, (3) retention rates, and (4) noncompliance and adherence rates (Table 3). A mixed methods approach will be used to assess acceptability through consideration of participants’ subjective ratings of the intervention content and modality of delivery and whether it was deemed enjoyable, understandable or clear, and beneficial, along with the measurement of motion sickness, system usability, and end-user perspectives on being fully present in the 3D virtual space. This will be accompanied by open-ended qualitative inquiry on overall thoughts across VR environments and associated content.

#### Aim 3: Testing of Intervention Effects

Participants will complete survey measures at 4 time points: baseline, immediately after the intervention (5 weeks after baseline), 3 months after the intervention, and 6 months after the intervention. Our exploratory aim will test intervention effects on depressive symptoms, positive psychological well-being (eg, optimism and positive affect) and distress, quality of life, disease-related biomarkers, missed hemodialysis treatment time (in minutes), and rates of hemodialysis sessions missed and hospitalizations.

### Central Hypothesis

Our central hypothesis is that our VR-based intervention will lead to postintervention improvements in depressive symptoms, emotional well-being, quality of life, and treatment adherence, along with reduced hospitalization rates and improvements across multiple clinical biomarkers. We will use repeated measures analysis of covariance to compare mean differences in depressive symptoms between the control and intervention arms as measured at baseline and follow-up, that is, immediately after the intervention at 5 weeks, and at 3 months and 6 months after the intervention. Analyses will be adjusted for baseline outcome values to account for the regression to the mean effect and to improve statistical power. To be precise, the average postrandomization effect in the VR-based intervention will be compared with the average postrandomization effect in the active control group. We will also implement an intention-to-treat approach. Multiple imputation procedures will be used across missing values to ensure inclusion of all observations, particularly those resulting from participants who withdraw, are lost to follow-up, or do not complete all assessments. Furthermore, sensitivity analyses will be implemented via the application of mixed effects models, assuming that data are missing at random. The independent variables include a time variable *t* (*t*=T0, baseline; T1, immediately after the intervention at 5 weeks; T2, six months after the intervention; and T3, six months after the intervention), a dummy variable *k* (*k*=1 if VR-based intervention condition and *k*=0 if active control arm), and baseline score of dependent variables. Unbalanced variables (mean differences across treatment arms) will be adjusted for, if necessary. Similar analyses will be repeated when examining outcomes of psychological well-being, quality of life, disease-related biomarkers, dialysis treatment adherence, and hospitalization rates.

At the individual level, we will calculate reliable change indices (RCIs) for each participant, as per Jacobson and Truax [49]. The RCI addresses whether the changes that participants experience are statistically reliable by comparing pre-post change with the reliability of the measurement instrument. RCI scores are calculated as follows:


RCI = *x*_2_
*− x*_1_ / *SE_diff_* **(1)**

where *x*_1_ represents a participant’s baseline score and *x*_2_ represents a participant’s postintervention score, and *SE_diff_* represents the SE of the difference between the 2 scores. RCI scores of >1.96 (approximately 2 SDs from the mean difference) are considered to be reliable [49]. We will present the percentage of participants who experienced reliable change for scores across the measures of interest. RCIs will also be expressed as percentage change from baseline by dividing the RCI score by the baseline score and multiplying by 100. At the sample level, we will conduct 1-tailed paired sample *t* tests to determine the magnitude of pre-post changes across the main outcomes of interest at each time point. In addition to reporting nominal *P* values, we will implement a false discovery rate approach to accommodate potential inflation of type I error rate owing to multiple comparisons. Furthermore, 95% CIs will be constructed to report meaningful differences between the groups, even if statistical difference is not achieved.

Sex as a biological variable will be addressed in exploratory analyses by tabulating disaggregated data by sex and via sex-stratified analyses of the main outcomes.

## Results

This RCT testing our *Joviality* software is funded by the National Institute of Diabetes and Digestive and Kidney Diseases through the funding period lasting from September 1, 2021, to July 31, 2024. The first 18 months of the funding period were dedicated to software development and the final build of the 3D immersive experience. The recruitment of patients on hemodialysis is scheduled to commence in June 2023. Participant recruitment is projected to end in December 2023, with the commencement of data analysis and submission of results for peer review in 2024.

## Discussion

### Overview

A dialysis treatment session is an ideal time for the delivery of strength-based interventions to boost psychological well-being because it constitutes a 3- to 4-hour window where patients are a captive audience [50], particularly because the lengthy treatment session is often described by patients as a wasteful and monotonous experience in a sterile environment. VR not only offers interactive technology to deliver much needed strengths-based interventions, but it also has the added advantage of allowing individuals on hemodialysis to digitally travel outside the clinical walls and into more relaxing and enjoyable settings. It has become evident that large-scale dissemination and scalability of face-to-face delivery of psychosocial interventions by trained clinicians are difficult, given the limited resources, unequitable health care access, and lack of bilingual practitioners, which makes the use of technology a particularly viable and attractive modality.

Our protocol provides the description of an RCT that adapts and tailors VR technology for use in a clinical population that encounters special needs and challenges. Our team of engineers has designed an intuitive system that requires simple head movement for interactability and that incorporates user-friendly features and an interface for adults with limited technology-based experience [51]. The trial sets the stage for fully immersive technologies in individuals with ESKD aimed at promoting positive emotional and physical health outcomes. Our trial is a preliminary step toward the design of patient-centered interactive VR technologies where patients are given autonomy to select their avatar, language, setting of choice, and much more. This, of course, would be accompanied by future integration of artificial intelligence to autonomously modify and predict end-user preferences for aesthetics, VR content, and travel locations.

VR technology has largely been exploited in video gaming and simulation training with clinicians and military personnel [52]. More recently, VR technology has been used in clinical settings with patients experiencing high-level pain and psychological phobias with favorable results [30,53]. In a randomized trial with patients with burn injuries aged 5 to 18 years, significantly lower pain scores were reported among those engaged in VR gaming while their wounds were being redressed [29]. In individuals with severe phobias, a systematic review found VR exposure therapy to be just as effective as in vivo therapies, particularly for cases of acrophobia (the fear of heights), claustrophobia, and blood injection phobia (the fear of injection exposure), with significant reductions in levels of fear, anxiety, and avoidance [54]. Other successful use cases with meta-analytic evidence include VR in palliative care [55], physical therapy and rehabilitation [56], and cognitive functioning (eg, memory and attention) [57].

In the context of hemodialysis, gamification techniques have focused primarily on nonimmersive gaming devices (eg, *Nintendo Wii*), which overlooks the advantages of fully immersive technologies where end users are teleported to artificial environments to interact with 3D virtual content. As VR technologies continue gaining traction in health care, it is important to decipher the mechanism involved and identify the active ingredients. Theorized pathways on the impacts of VR on health may include (1) cognitive distraction through appealing stimuli, (2) decreased autonomic parameters and healthful physiological responses, (3) targeted induction of body movement and physical activity, and (4) the delivery of evidence-based programming. Much research remains to be conducted to fully understand the role of VR in health care and the mechanisms at play. However, certain considerations should be kept at the forefront, including accessibility across the spectrum of socioeconomic backgrounds, so that immersive technology does not further widen the chasm of health disparities in the United States and abroad.

Our previous work, using the largest cohort of Hispanic or Latino adults, that is, the Hispanic Community Health Study/Study of Latinos, found a positive association between psychological well-being and ideal cardiovascular health in adults with early-stage chronic kidney disease [58]. Each unit increase in positive affect was associated with higher odds of ideal cardiovascular health across multiple indicators. There is no doubt that accumulating evidence documents the merit and healthful benefits of positive emotions and psychological assets in the context of disease-related stress and trauma. As such, this VR-based trial is also a call for greater focus on the strengths and factors of resilience that individuals with ESKD possess, with a focus on programming that will further amplify these positive attributes.

### Potential Limitations and Conclusions

Despite the innovativeness and strengths of this RCT, a few limitations should be noted. First, the inclusion criteria capturing elevated symptoms of depression will use the self-report BDI-II survey measure and will not rely on a more precise clinical diagnosis. Nonetheless, the BDI-II has been widely tested in individuals on hemodialysis and has documented satisfactory psychometric properties to suggest a higher likelihood of clinical depression. Generalizability of the results may be restricted if recruitment fails to enroll individuals on hemodialysis who are averse to the use of technology or if those with severe depression are reluctant to engage with research staff, thus selectively reducing enrollment rates. It is also possible that the VR content may lead to relaxation that induces sleep, a possibility that our engineering team has contemplated. Thus, triggering design features have been incorporated to alert research staff and end users if prolonged inactivity in the VR space is detected. Finally, given the small targeted enrollment goal, we may not have the power to explore mechanisms through which VR exerts protective effects (if any), nor will it be possible to fully test important effect modifiers of the intervention (eg, race, ethnicity, and socioeconomic status).

In conclusion, we seek to examine whether VR is an effective platform to deliver a positive psychological intervention in individuals on hemodialysis with comorbid depression to improve emotional well-being and clinical health outcomes. If successful, VR headsets could be salient clinical tools in hemodialysis clinics in the United States and abroad. Our VR-based approach could become the new evidence-based tool that clinics house and adopt to improve mental health outcomes in individuals on hemodialysis, with the added potential benefit of improving treatment adherence and healthy longevity.

## Abbreviations

BDI-II: Beck Depression Inventory-II
CONSORT: Consolidated Standards of Reporting Trials
CONSORT-SPI 2018: Consolidated Standards of Reporting Trials 2010 statement for social and psychological interventions
EMR: electronic medical record
ESKD: end-stage kidney disease
HIPAA: Health Insurance Portability and Accountability Act
HMD: head-mounted display
ICD-10: International Classification of Diseases, Tenth Revision
PROMIS: Patient-Reported Outcomes Measurement Information System
RCI: reliable change index
RCT: randomized controlled trial
REDCap: Research Electronic Data Capture
VR: virtual reality

## Data Availability

This paper presents details of the protocol and design for a randomized controlled trial testing virtual reality in the context of hemodialysis; as such, this study does not generate or analyze an associated data set, making data sharing nonapplicable. Once the protocol is implemented and data are made available, we will use a National Institutes of Health–approved data repository to share derived data in a deidentified format and follow rigorous procedures to ensure protection of the privacy, rights, and confidentiality of human research participants. Finally, the public will have access to the detailed protocol and general findings of the trial (when available) at ClinicalTrials.gov (NCT05642364).
